# Mental health nursing care for people with diabetes mellitus: An integrative review *

**DOI:** 10.1590/1518-8345.6827.4074

**Published:** 2023-12-04

**Authors:** Bianca Brandão da Silva, Maria Helena de Melo Lima, Maria Giovana Borges Saidel

**Affiliations:** 1 Universidade Estadual de Campinas, Faculdade de Enfermagem, Campinas, SP, Brasil.; 2 Becaria de la Coordenação de Aperfeiçoamento de Pessoal de Nível Superior (CAPES), Brasil.; 3 Becaria del Conselho Nacional de Desenvolvimento Científico e Tecnológico (CNPq), Brasil.

**Keywords:** Diabetes Mellitus, Diabetes Complications, Mental Health, Nursing Care, Self-Management, Nurses’ Practice Patterns, Diabetes Mellitus, Complicaciones de la Diabetes, Salud Mental, Atención de Enfermería, Automanejo, Pautas de la Práctica en Enfermería, Diabetes Mellitus, Complicações do Diabetes, Saúde Mental, Cuidados de Enfermagem, Autogestão, Padrões de Prática em Enfermagem

## Abstract

**Objective:**

evaluate the evidence available on mental health nursing care for people with diabetes mellitus at different levels of health care.

**Method:**

integrative literature review. The search was conducted in five databases. The sample consisted of 14 studies. The studies were exported to the EndNote manager and their data to a Microsoft Excel spreadsheet. The methodological quality of the studies was evaluated using tools proposed by the Joanna Briggs Institute. Sampling, categorization, evaluation, interpretation of the results, and synthesis of the included studies were carried out by two reviewers independently. The descriptive analysis of the results is presented in three categories.

**Results:**

self-care guidelines enhanced by the social support network, encompassing physical and psychological tools and strategies; therapeutic communication and psychotherapy strategies, focusing on psychotherapy and therapeutic communication; and self-management interventions, addressing self-care based on behavioral theories.

**Conclusion:**

the synthesis of knowledge revealed that guidelines for self-care enhanced by the social support network, psychotherapy and therapeutic communication strategies, and self-management interventions are positive interventions that contribute to people with mental disorders and diabetes mellitus in the prevention of diseases.

## Abstract (es)

**Objetivo:**

evaluar las evidencias disponibles en la literatura sobre la atención de enfermería en salud mental a personas con diabetes mellitus en los diferentes niveles de atención de salud.

**Método:**

revisión integrativa de la literatura. Búsqueda en cinco bases de datos. Muestra de 14 estudios, exportados al gerenciador *EndNote*®. Los datos se organizaron en planilla de *Microsoft Excel*®. La calidad metodológica de los estudios fue evaluada mediante herramientas propuestas por el *Joanna Briggs Institute*. Las etapas de muestreo, categorización, evaluación, interpretación de resultados y síntesis de los estudios incluidos fueron realizadas por dos revisores de manera independiente y ciega. El análisis descriptivo de resultados está presentado en tres categorías.

**Resultados:**

recomendaciones para autocuidado reforzado por la red de apoyo social, incluyendo herramientas y estrategias de los ámbitos físico y psíquico; estrategias de comunicación terapéutica y psicoterapia, haciendo foco en la psicoterapia y la comunicación terapéutica; e intervenciones de autogestión/autogerenciamiento, abordando el autocuidado en base a teorías conductuales.

**Conclusión:**

la síntesis de conocimientos reveló que las pautas de autocuidado reforzadas por la red de apoyo social, las estrategias de comunicación terapéutica y psicoterapia y las intervenciones de autogestión/autogerenciamiento son intervenciones positivas que ayudan a las personas con diabetes mellitus y trastornos mentales a prevenir complicaciones.

## Abstract (pt)

**Objetivo:**

avaliar as evidências disponíveis sobre os cuidados de enfermagem em saúde mental para pessoas com diabetes *mellitus* nos diferentes níveis de atenção à saúde.

**Método:**

revisão integrativa da literatura. Busca realizada em cinco bases de dados. Amostra composta por 14 estudos. Os estudos foram exportados para o gerenciador *EndNote*, e seus dados, para uma planilha desenvolvida pelo *Microsoft Excel*. A qualidade metodológica dos estudos foi avaliada por meio de ferramentas propostas pelo *Joanna Briggs Institute*. Etapas de amostragem, categorização, avaliação, interpretação dos resultados e síntese dos estudos incluídos foram realizadas por dois revisores de forma independente e mascarada. A análise descritiva dos resultados é apresentada em três categorias.

**Resultados:**

orientações para o autocuidado potencializadas pela rede de suporte social, englobando ferramentas e estratégias no âmbito físico e psíquico; estratégias de comunicação terapêutica e psicoterapia, focalizando a psicoterapia e a comunicação terapêutica; e intervenções de autogestão/ autogerenciamento, abordando o autocuidado com base em teorias comportamentais.

**Conclusão:**

a síntese do conhecimento revelou que as orientações para o autocuidado potencializadas pela rede de suporte social, estratégias de comunicação terapêutica e psicoterapia e intervenções de autogestão/autogerenciamento são intervenções positivas que auxiliam as pessoas com transtornos mentais e diabetes *mellitus* na prevenção de agravos.

**Highlights:**

**(1)** The Brazilian literature on mental health care for people with diabetes is scarce.

**(2)** The review addresses mental health care for people with diabetes mellitus.

**(3)** Self-care enhanced by the support network is reflected in mental health.

**(4)** Therapeutic communication and behavioral psychotherapy are effective treatments.

**(5)** Interventions based on self-management reduce psychological suffering.

## Introduction

Mental health care should be considered an essential part of care for people with diabetes mellitus (DM) ^(^ 1 ^)^. Mental disorders (MDs) and feelings of psychological distress, which differ conceptually in terms of duration, severity of symptoms, and impact on the individual’s functionality, are common in people with DM ^(^ 2 ^-^ 4 ^)^. In addition, depression and anxiety are highly prevalent MDs, ranging from 18% to 54.3% in people with DM ^(^ 4 ^-^ 7 ^)^. Scientific evidence points to a linear association between depressive symptoms and DM ^(^ 7 ^-^ 9 ^)^. As a result of this scenario, unfavorable conditions arise, diabetes self-management is impaired, metabolic control worsens ^(^ 10 ^)^, the incidence of microvascular and macrovascular complications increases, and life expectancy decreases ^(^ 1 ^,^ 10 ^)^. The main complications of DM can contribute directly or indirectly to worsening conditions related to the musculoskeletal system, digestive system, cognitive function, and mental health ^(^ 6 ^,^ 11 ^)^. In this sense, these complications can cause psychological distress that can lead to MDs if mental health care is not provided early on ^(^ 1 ^,^ 12 ^)^. This condition impacts the daily lives of these people in individual, family, and community settings, and they can experience severe physical and emotional restrictions ^(^ 3 ^,^ 13 ^)^. The complexity of the scenario requires early structured mental health care, with an emphasis on psychosocial needs ^(^ 14 ^-^ 15 ^)^. 

Estimates are that by 2045, the population of South and Central America (SACA) with DM will be 49 million, leading to a 25% increase in the prevalence of DM, reaching 11.9%. In Brazil, data from the 2021 survey showed a prevalence of 10.5% of adults aged between 20 and 79 with DM. This situation is a major challenge for public health, given that in 2021, 65.3 billion dollars were spent on DM in the SACA region, representing 6.7% of the total global expenditure ^(^ 16 ^)^. 

Given this reality, the provision of care in health teams can enable interventions based on evidence-based clinical practices, allowing existing structures to be strengthened with the aim of offering integrated and continuous care ^(^ 14 ^,^ 17 ^)^. The search for better therapeutic results in the modern health system is challenging. Current health care is divided by specialty, and this reality makes care fragmented ^(^ 1 ^)^. People with DM and MDs are not assisted according to the principle of comprehensiveness, as they are usually cared for by different teams that offer care for only one of the morbidities ^(^ 3 ^)^. As far as the nursing team is concerned, nursing care stands out in health promotion, disease prevention, and health recovery and rehabilitation, which means that caring for patients with DM and MDs requires nursing professionals to take a broad view of the needs imposed by the disease. This process corroborates the provision of continuous care in the management of chronic conditions and their complications, playing a fundamental role in the structuring of health education processes ^(^ 17 ^)^. An ideal model for patients with DM and MDs would consist of an integrative health approach, consisting of educational activities and mental health care, leading to an improvement in MD symptoms and increased adherence to the proposed therapies ^(^ 1 ^,^ 17 ^)^. 

Given the above, it is essential to learn about mental health care for people with chronic conditions, such as DM, to achieve better results in controlling the disease. In addition, nurses are responsible for planning and implementing nursing care, to improve adherence to treatment, prevent complications, or detect them early, to effectively help the patient’s well-being. Therefore, this study aims to evaluate the evidence available on mental health nursing care for people with DM at different levels of health care.

## Method

### Study design

An integrative review (IR) of the literature was developed according to the following phases: drafting the review question, searching the literature for primary studies, evaluating the primary studies, analyzing the data, and presenting the review ^(^ 18 ^)^. The IR protocol was registered in the UNICAMP Research Data Repository on December 6 ^th^, 2022, and is available from: https://doi.org/10.25824/redu/CXJHTW. 

### Setting

The study was carried out in the municipality of Campinas, state of São Paulo, Brazil.

### Period

The study period was from January 2021 to January 2023.

### Population

The review question was: What evidence is available in the literature on mental health nursing care for people with DM at the different levels of health care? Based on the acronym PICo ^(^ 19 ^-^ 20 ^)^, where P (Population): People with DM with or without a medical diagnosis of MD; I (Interest): Mental health care/nursing care; and Co (Context): Levels of health care, i.e. patients who are in a follow-up/accompaniment the health network. This acronym helps to carry out an effective search based on the elaboration of the review question to guide the research according to the proposed objectives ^(^ 20 ^)^. 

### Selection criteria

The eligibility criteria for the development of the IR were: primary studies with no time restriction, whose authors investigated nursing care in mental health for people with diabetes mellitus type 1 (DM1) or diabetes mellitus type 2 (DM2), with or without a medical diagnosis of MD, at the different levels of health care, available electronically in full, published in English, Spanish or Portuguese. The exclusion criteria were: documents in letter format, editorials, single case studies, books, theses, review articles, and articles not available in full. The flowchart for identifying the studies found was drawn up following the Preferred Reporting Items for Systematic Reviews and Meta-Analyses (PRISMA) guidelines ^(^ 21 ^)^. 

### Sample definition

The following electronic databases/websites were used: PubMed, Web of Science, Scopus, LILACS, SciELO, CINAHL, and the American Psychological Association. The search strategy was built using the Health Sciences Descriptors (DeCS) and the terms indexed in the Medical Subject Headings (MeSH), with the help of the Boolean operators AND and OR, aiming for high sensitivity in each database and a wide range of results. The terms used were: “Diabetes Mellitus”, “Nursing Care”, “Nursing”, “Nursing Services”, “Mental Health”, “Mental Disorders”, “Psychiatric Nursing”, “Mental Health Services”, “Psychiatric Nursing” and “Health Care Levels”.

During the searches, we opted for the strategy of crossing descriptors and keywords using Boolean operators, so initially, the descriptors of each set of the PICo strategy were combined using the OR or AND Boolean connector and then each set was combined with the AND connector ( Figure 1). 

**Figure 1 -:** Search strategy used. Campinas, SP, Brazil, 2023

Objective/ Problem	What evidence is available in the literature on mental health nursing care for people with diabetes mellitus?		
	Population	Interest	Context
Extraction	People with mental disorders and diabetes mellitus	Mental health nursing/Nursing care	Health care levels
Conversion	People with mental disorders and diabetes mellitus	Mental health nursing/Nursing care	Health care levels
Combination	Mental Disorders; Diabetes Mellitus	Mental Health Care; Nursing Care; Nursing; Nursing Services; Mental Health; Mental Health Services; Psychiatric Nursing	Health care levels
Construction	“Mental Disorders” AND “Diabetes Mellitus”	“Nursing Care” OR “Nursing” OR “Nursing Services” OR “Mental Health” OR “Psychiatric Nursing” OR “Mental Health Services”	“Health care levels”
Use	(“Mental Disorders” AND “Diabetes Mellitus”) AND (“Nursing Care” OR “Nursing” OR “Nursing Services” OR “Mental Health” OR “Psychiatric Nursing” OR “Mental Health Services”) AND (“Health Care Levels”)		

The articles found were exported to the online reference manager EndNote (Clarivate Analytics), allowing for storage and organization, as well as checking and deleting duplicate records ^(^ 22 ^)^. The article selection stage was carried out independently by two researchers, whose differences were resolved by consensus. The studies were selected in two stages: 1) reading the titles and abstracts according to the inclusion and exclusion criteria and 2) reading the full article. 

### Data collection

To collect and categorize data from the studies included in the review, a spreadsheet was developed using Microsoft Excel software, version 2013, recording the following information: authors, year of publication and country of affiliation, title of the article, objective, methodology, level of evidence, characterization of the sample/participants, interventions, main results, and conclusion. The stages of screening, selection, and analysis of the articles were carried out independently by two researchers, whose differences were resolved by consensus.

### Data analysis

The following classifications were considered for the levels of evidence: I, evidence obtained from a systematic review or meta-analysis of relevant randomized controlled clinical trials; II, derived from a randomized controlled trial with individuals randomized to a treatment/control group; III, derived from a non-randomized controlled trial, not randomized to a treatment/control group; IV, obtained from a case-control or cohort study; V, derived from a systematic review of qualitative or descriptive studies; VI, derived from a qualitative and descriptive study; VII, derived from the opinion or consensus of a committee of experts ^(^ 23 ^)^. 

The selected studies were analyzed and synthesized through critical reading and descriptive content grouping, classifying the studies into categories for later discussion, based on the available scientific literature on the subject. They were allocated into three categories of care that emerged from the primary studies, which was considered fundamental for discussing the findings. This process took place with the help of a spreadsheet developed using Microsoft Excel software.

## Results

**Figure 2 -:**
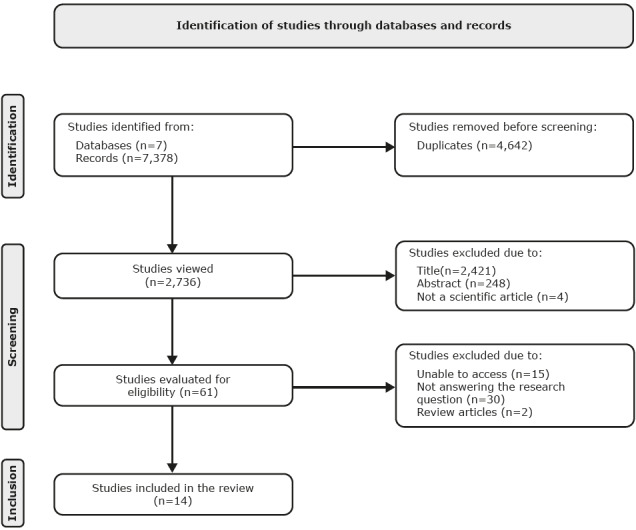
Flowchart of the process of selecting the primary studies included in the integrative review according to the Preferred Reporting Items for Systematic Review and Meta-Analyses (PRISMA). Campinas, SP, Brazil, 2023

The results of this IR correspond to the analysis of 14 published scientific articles, selected according to the PRISMA guidelines ( Figure 2). 

Given the results, it was possible to identify different types of mental health care that were addressed in the studies in this sample. From a qualitative point of view, to better understand and organize the results, they were divided into three thematic categories: “Self-care guidelines enhanced by the social support network”, “Therapeutic communication and psychotherapy strategies” and “Self-management interventions”. The categories, the details of the analysis, and the number of studies that were allocated to each category are shown in Figure 3. 

**Figure 3 -:** Thematic categories. Campinas, SP, Brazil, 2023

Thematic categories	Analysis details	Number of studies in the thematic category
1. Self-care guidelines enhanced by the social support network	Studies including tools and strategies in the physical and psychological spheres	4
2. Therapeutic communication and psychotherapy strategies	Studies that gathered information on care such as psychotherapy and therapeutic communication in individual and group settings	6
3. Self-management interventions	Studies addressing the improvement of self-care based on behavioral theories	6

The time span of the studies analyzed corresponds to the period from 2009 to 2022, with 21.43% (n=3) of them concentrated in 2020. The 14 studies were published in national (n=1) and international (n=13) journals. As for the origin of the studies, 21.43% (n=3) are from China, 21.43% (n=3) from the United States of America and the rest (7.14%) from the United Kingdom, Canada, Brazil, The Netherlands, Indonesia, Turkey, Iran, and Norway, with one study each, respectively. Regarding the levels of health care at which the studies were carried out, 46.86% (n=6) were conducted in primary care, 28.57% (n=4) in secondary care, and 21.43% (n=3) in tertiary care institutions. The category of levels of care did not apply to one of the studies (7.14%) ( Figure 4). 

Regarding the study design, 35.71% (n=5) were qualitative, 28.57% (n=4) randomized clinical trials, 7.14% (n=1) quasi-experimental randomized with a control group, 7.14% (n=1) cross-sectional descriptive, 7.14% (n=1) descriptive correlational, 7.14% (n=1) observational randomized, and 7.14% (n=1) non-randomized clinical trial ( Figure 4). As for the levels of evidence, 21.43% (n=3) had level I, 21.43% (n=3) level II, 50.00% (n=7) level VI, and 7.14% (n=1) level VII ( Figure 4). 

**Figure 4 -:** Characterization of the articles included in the integrative review: Primary study, year, country of origin, design, level of health care, outcomes/conclusions, level of evidence, and database. Campinas, SP, Brazil, 2023

Primary study/Year/ Country of origin	Study design/Level of health care	Outcomes/Conclusions	Level of evidence	Database
Arifin, et al. 2020/ Indonesia ^(^ 24 ^)^	Qualitative study/Primary care	Spirituality and acceptance strategies are common coping mechanisms to reduce stress and suffering. It was revealed that individuals had a generally positive attitude towards treatment, as well as a greater demand for information about DM.	VI	Web of Science
Blixen, et al. 2018/United States ^(^ 25 ^)^	Qualitative study/Primary care	Educational processes led by nurses and carried out through evidence-based interventions (Targeted Training in Illness Management - TTIM) provide a space for peer support. This situation has strengthened self-management practices and the building of strategies to minimize the morbidity and mortality of groups with DM and MDs.	VI	Scopus
Collins-McNeil, et al. 2009/United States ^(^ 26 ^)^	Descriptive correlational study/Primary care	Physical exercise, weight loss, and a social support network were identified as protective measures for African-American women with DM and depression. It is worth reinforcing that additional social support contributes to lifestyle changes for better self-management of DM.	VI	PubMed
Ince, et al. 2017/Turkey ^(^ 27 ^)^	Qualitative study/ Secondary care	The participants had difficulties with self-care related to DM. However, participants with DM and MDs presented more challenges in managing DM due to symptoms of psychological distress, adverse effects of psychotropic drugs, and less knowledge about DM. The results showed better social interaction and exchange relationships, construction of a collective identity, and collaborative and coping capacity.	VI	PubMed
Ismail, et al. 2018/United Kingdom ^(^ 28 ^)^	Randomized clinical trial/ Primary care	Training nurses in Motivational Interviewing (MI) and Cognitive Behavioral Therapy (CBT) with the aim of supporting DM self-management did not cause improvements in glycemic control when compared to standard care.	I	PubMed
Kaboudi, et al. 2017/Iran ^(^ 29 ^)^	Experimental study with control group/Secondary care	Treatment based on acceptance and commitment practices in mental health, together with medication and therapy, was positive as a complementary care strategy to improve the mental health and the general condition of people with DM.	II	PsycINFO
Karlsen, et al. 2012/ Norway ^(^ 30 ^)^	Cross-sectional study/ Not applicable	The improvement in the perceived social support network contributed to the remission of suffering, although it did not influence metabolic control.	VII	Scopus
Lawless, et al. 2016/United States ^(^ 31 ^)^	Randomized clinical trial/ Primary care	TTIM led by nurse educators provided self-management skills and increased adherence to treatment in people with severe DM and MDs.	II	Scopus
Meeuwissen, et al. 2011/Netherlands ^(^ 32 ^)^	Non-randomized clinical trial/Primary care	The implementation of screening based on a ‘self-help intervention’ and carried out by DM nurse educators contributes to the early identification of MDs (anxiety and depression) which complicates DM treatment.	II	Scopus
Oliveira, et al. 2011/Brazil ^(^ 33 ^)^	Qualitative study/ Secondary care	The identification of feelings and perceptions associated with the diagnosis of DM through group exploration sessions revealed different strategies, barriers, and levels of family social support. These differences in the perception of DM should be considered as they can influence treatment adherence and establish components that should be taken into account when planning care.	VI	Web of Science
Stenov, et al. 2020/Canada ^(^ 34 ^)^	Qualitative study/ Secondary care	MDs impaired good glycemic control and interfered with DM treatment, given the difficulty of maintaining the care routine alongside the symptoms of MDs, in addition, there was little dialogue with health professionals on the subject. The individualized interventions helped to support people with MDs in self-managing their DM.	VI	Web of Science
Wu, et al. 2020/China ^(^ 35 ^)^	Randomized observational study/ Tertiary care	Integrated and shared mental health care such as psychotherapy, pharmacotherapy, and self-management education should be offered to people with severe DM and MDs. These measures indicated protection from feelings of psychological distress or worsening of existing MD conditions.	VI	Web of Science
Li, et al. 2022/China ^(^ 36 ^)^	Randomized clinical trial/ Tertiary care	Educational activities aimed at self-management and psychological intervention relieved negative emotions in people with DM, stabilized glycemic indices, and improved quality of life, showing good potential for clinical promotion.	I	Scopus
Yao, et al. 2021/China ^(^ 37 ^)^	Randomized clinical trial/ Tertiary care	Behavioral interventions associated with the Motivation Theory promoted improved resilience in people with DM, with reduced levels of depression, improved quality of life, and lower glycemic levels.	I	PubMed

## Discussion

The findings are presented and discussed in the three aforementioned thematic categories: Self-care guidelines enhanced by the social support network, Therapeutic communication and psychotherapy strategies, and Self-management interventions.

### Self-care guidelines enhanced by the social support network

The literature has shown that guidance for self-care practices is a type of care that reverberates in people’s mental health. The studies analyzed in the IR indicated the need for educational guidance on practices aimed at self-knowledge and providing tools to promote self-care. The social support network of people with DM can boost self-care guidelines and these actions promote mental health ^(^ 24 ^,^ 26 ^-^ 27 ^,^ 30 ^)^. Emphasizing that engaging in good self-care practices, such as adopting a healthy eating plan, exercising regularly, managing blood glucose, and maintaining adherence to medication therapy, is fundamental to reducing the long-term risks of the chronic condition ^(^ 28 ^)^ as well as reducing treatment costs and, consequently, minimizing anxious states and other feelings of psychological distress ^(^ 38 ^)^. 

In this sense, people with MDs and DM need to experience symptoms about both chronic conditions, presenting different challenges in the management of diabetes, due to mental symptoms and adverse effects of psychotropic medications. Therefore, MDs together with DM can be unfavorable in self-care practices ^(^ 27 ^)^ - as many symptoms of mental disorders interfere with will, determination, and functional activities ^(^ 6 ^)^ - requiring careful monitoring by the health team ^(^ 27 ^)^. This statement should alert nurses, who are usually involved in embracement, initial assessment, or triage activities in primary health care services, to consider strategies for monitoring and implementing effective care for the conditions mentioned. 

Although there are worse clinical outcomes in people with DM and MDs, when there is a social support network, patients have better levels of self-efficacy related to DM, which has been associated with improved glucose levels and greater engagement in self-management of the disease ^(^ 5 ^,^ 7 ^,^ 9 ^)^. A study has shown that improving the social support network perceived by people with DM causes a regression in feelings of distress ^(^ 30 ^)^. Therefore, initiatives on this subject should be part of mental health care practices to help people cope with the difficulties presented by both chronic conditions. Thus, mapping the social support network using tools can contribute and broaden people’s perception of the importance of self-care ^(^ 27 ^)^. 

Encouraging behavior and positive feedback from health professionals can be considered motivating factors for these people to carry out self-care practices ^(^ 27 ^,^ 38 ^)^. In this sense, the relevance of systematized nursing consultations stands out, to continuously build an individualized care plan, in order to promote management of care, encourage therapeutic and adherence to both diseases. This building process should include biopsychosocial issues inherent to the human person, as well as seek social support ^(^ 17 ^,^ 27 ^)^. 

### Therapeutic communication and psychotherapy strategies

Mental health care based on therapeutic communication and psychotherapy was the most significant result in terms of the number of articles. The interpersonal relationship using therapeutic communication strategies appears as a possibility of mental health care for patients with MDs. Individual and group psychotherapy anchored in theoretical frameworks, with emphasis on CBT, MI, and psychoanalysis, emerged as a powerful tool ^(^ 28 ^-^ 29 ^,^ 32 ^-^ 34 ^,^ 39 ^)^. 

Regarding mental health care focused on a relational field, of which therapeutic communication and psychotherapies stand out, the evidence shows that these can promote and improve mental health by intensifying motivation to self-manage comorbidities. In other words, they offer a greater chance of building autonomy and responsibility in treatment ^(^ 10 ^,^ 40 ^)^. 

This care can be provided in group ^(^ 33 ^)^ or individual settings and is generally used as a supportive therapy ^(^ 32 ^)^, i.e. in conjunction with other therapies, including medication. A randomized clinical trial conducted in the UK involved CBT and MI conducted by qualified nurses, in which there were no significant differences in results or improvement in glycemic levels ^(^ 28 ^)^. Another clinical study conducted by nurses using guided self-help intervention (supportive therapy) showed positive results with a significant reduction in symptoms of anxiety and depression ^(^ 32 ^)^. In addition, psychotherapy sessions based on the acceptance and commitment therapy in mental health, combined with drug treatment, were shown to improve the mental health and overall mental state of people with DM ^(^ 29 ^)^. 

The studies involve themes that can be addressed in these mental health care strategies: Reflections on self-esteem related to the functional losses caused by DM; adaptive skills of the individual through the exploration of interpersonal behaviors ^(^ 39 ^)^ ; identification of individual solutions through the therapeutic relationship in spaces focused on mental health care ^(^ 34 ^)^ ; values and beliefs coordinated with behavioral responses ^(^ 33 ^)^. 

Cognitive behavioral therapy for people with DM2 has been shown in other studies to be effective and potentially cost-effective when combined with first-line drug treatments, physical exercise, and a proper diet plan ^(^ 11 ^,^ 40 ^)^. 

Other studies have shown that the combination of mental health care based on therapeutic communication and psychotherapy with self-care practices and drug therapy results in an improvement in depressive symptoms in people with DM. These studies have described a greater and longer-lasting positive effect on depressive symptoms than when treatment is done with antidepressants alone ^(^ 12 ^,^ 15 ^,^ 40 ^)^. 

Finally, it should be emphasized that the mental health care planned and operationalized by the nursing team is based on the therapeutic relationship and the theoretical framework that this technique needs in order to be applied effectively ^(^ 41 ^)^. 

### Self-management interventions

Self-management interventions were highlighted in this review as evidence of mental health care. The fact that the person with DM achieves autonomy in their treatment and positive results in glycemic levels and other self-management indicators seems to reduce MD symptoms and feelings of psychological distress ^(^ 25 ^,^ 31 ^,^ 35 ^,^ 42 ^)^. 

The concept of self-management refers to the person’s ability to manage the clinical and psychosocial consequences, along with lifestyle changes, in relation to living with a chronic condition, in this case DM and an MD, and this process is mediated by the team in the different health facilities ^(^ 42 ^)^. This mental health care can provide autonomy and, at the same time, bring the person’s co-responsibility into the treatment context, broadening the understanding that care requires effective adherence to achieve good outcomes. 

For clinical nursing practice, self-management interventions should be aimed at encouraging and improving self-care, as they can bring benefits and promote the person’s autonomy to manage DM and MDs. This care is pointed out in the literature as enhancing engagement in treatment, in the learning process, and improving overall well-being ^(^ 35 ^)^. This mental health care is presented in a complex way, since it involves planning aspects related to achieving objectives, intensity, duration, environment, mode of operation (group or individual), type, and training of the health professional or patient ^(^ 32 ^,^ 43 ^-^ 44 ^)^. 

Self-management is influenced by behavioral psychology and based on theories of behavior change in health, such as social cognitive theory and the theory of rational action and planned behavior, among others. During this intervention, health professionals work with the individual on their drug therapy, healthy eating, physical activity, monitoring glucose levels, and regular appointments ^(^ 35 ^,^ 42 ^)^. In this context, it is important to emphasize the need for nurses to be equipped with mental health theoretical frameworks ^(^ 41 ^)^, through training aimed at early detection and more accurate and comprehensive interventions for people with DM and MDs. 

People with DM and some form of mental illness may have inadequate self-management for both conditions. This reality can result in blood glucose instability and subsequent complications, as well as physical, psychological, and social health challenges. All these variables cause a low state of functional health and unsatisfactory self-management ^(^ 35 ^)^. Early recognition of these aspects in this population, especially in facilities that are gateways to the health system, is fundamental ^(^ 12 ^)^. By recognizing this population, health professionals can intervene early, and this early mapping increases their capacity for self-management and management of the condition ^(^ 35 ^)^. 

The studies identified in this review addressed the complications of DM and how they are more likely to develop when there is inadequate self-management of this chronic condition. Mental health care based on self-management interventions should be adapted for people with severe 2DM and MDs in order to increase this capacity ^(^ 35 ^)^. 

The application of the TTIM intervention by nurses to empower people with DM and MDs can make people actively involved in their care ^(^ 31 ^)^. In addition to being considered a self-management tool, this intervention provides a broadening of health knowledge ^(^ 13 ^,^ 25 ^)^. 

Targeted training in illness management is an intervention that can be led by nurse educators and content “experts”, and is carried out in two stages consisting of weekly group sessions and a monthly follow-up, either in person or by telephone, over 48 weeks to support the personal care plan. Nurses address facilitators and barriers to care and provide health education on specific topics, in addition to coordinating communication with other professionals. Interaction between nurse educators, other professionals, and program participants is a key feature of this intervention ^(^ 31 ^)^. In a qualitative study, conducted using a randomized clinical trial, which included 10 people with DM and severe MD who participated in a TTIM self-management intervention, positive meaning was attributed to the group experiences, as well as increased knowledge about their health and increased self-confidence ^(^ 25 ^)^. This highlights the importance of health professionals, with an emphasis on nursing, developing accurate, evidence-based clinical practice for mental health care and DM management. 

Therefore, care should be guided by strategies aimed at mapping and evaluating this population early on and offering mental health care in order to mediate the management of DM and MD symptoms ^(^ 28 ^,^ 39 ^)^. These actions can promote physical and mental health, helping to improve overall well-being and reduce long-term risks ^(^ 34 ^-^ 35 ^,^ 45 ^)^. 

This IR was developed with the necessary methodological rigor in mind. However, some limitations were identified. Firstly, the criterion of including articles available in full and free of charge may not have allowed for the inclusion of studies pertinent to the proposal. In addition, some of the studies included have methodological limitations, including a fragile sample composition, a low level of evidence, and insufficient description of data collection, which, despite not impeding the IR, may have made the discussion of some studies more vulnerable. To remedy this limitation, future systematic reviews could be developed specifically considering studies with a higher level of scientific evidence. In addition, only one national primary study was identified that answered the research question, which may characterize a gap in this knowledge in the national context.

Given the above, mental health care for people with DM and MDs should be implemented by health professionals, with an emphasis on nursing professionals who are generally involved in embracement, screening, and individualized care activities, with a view to providing psychosocial support, a care plan for DM and MD self-management, and promoting prevention strategies for both health problems. By implementing this care, nurses will also be able to monitor feelings of psychological distress and promote mental health, as well as prevent complications.

Finally, it should be noted that the few qualitative studies identified a priori were more closely related to the subject of this review, which points to a gap in research involving nursing, diabetes, and mental health.

## Conclusion

The results of this IR showed that self-care guidelines enhanced by the social support network, therapeutic communication and psychotherapy strategies, and self-management interventions are positive interventions that help people with DM and MDs to prevent problems. Therefore, professionals, with an emphasis on nurses, should identify and map symptoms of psychological distress early on, so that mental health care is effective and can have a positive impact on engagement in DM treatment. However, the implementation of such care still requires the development of research with robust designs.
